# Data on the physical characterization of oil in water emulsions

**DOI:** 10.1016/j.dib.2016.08.038

**Published:** 2016-08-28

**Authors:** Aldana L. Zalazar, María F. Gliemmo, Carmen A. Campos

**Affiliations:** a Universidad de Buenos Aires, Facultad de Ciencias Exactas y Naturales, Departamento de Industrias, Buenos Aires, Argentina; b Consejo Nacional de Investigaciones, Científicas y Técnicas de la República Argentina, Argentina

## Abstract

This article contains experimental data and images for the physical characterization of oil in water emulsions. Mentioned data are related to the research article “Effect of stabilizers, oil level and structure on the growth of *Zygosaccharomyces bailii* and on physical stability of model systems simulating acid sauces” (A.L. Zalazar, M.F. Gliemmo, C.A. Campos, 2016) [1]. Physical characterization of emulsions was performed through the evaluation of Span and Specific Surface Area (SSA) determined by light scattering using a Mastersizer. Furthermore, microscopy images were recorded by confocal scanning laser microscopy (CSLM). The latter are presented to collaborate in the analysis of emulsion microstructure.

**Specifications Table**

**Table:** 

Subject area	*Physics, Chemistry.*
More specific subject area	*Food Chemistry*
Type of data	*Table and image*
How data was acquired	*Span and Specific Surface Area (2000 with a Hydro 2000 MU as dispersion unit, Malvern Instruments, Worcestershire, United Kingdom), confocal scanning laser microscopy image (Olympus confocal microscope FV 300).*
Data format	*Raw and analyzed*
Experimental factors	*Different oil in water emulsions were formulated varying oil levels and stabilizer agents.*
Experimental features	*Several emulsions were prepared by high speed homogenization. Span and Specific Surface Area (SSA) were obtained from the analysis of droplet size of emulsions and microscopy images were recorded.*
Data source location	*Departamento de Industrias, Facultad de Ciencias Exactas y Naturales, Universidad de Buenos Aires, Ciudad Universitaria, Buenos Aires, Argentina.*
Data accessibility	*Data are presented in this article.*

**Value of the data**

• Span and SSA data provide further information on the distribution of droplet size of emulsions.
• Span gives information on the polydispersity of the sample and SSA is associated with emulsion stability. Both parameters are useful to evaluate stabilizers action on emulsion stability.
• CSLM provides information about the size, concentration and organization of the droplets in an emulsion.
• For studying emulsions, microscopy provides more visual and direct information than other techniques-e.g. light scattering, nuclear magnetic resonance, and conductivity methods. In the case of CSLM, simple sample preparation with minimal alterations of the environmental conditions makes it very suitable to study phase stability.
• The data can be useful for other researchers investigating the effects of stabilizers, oil level on physical stability of emulsions.

## 1 Data

**Fig. 1:**
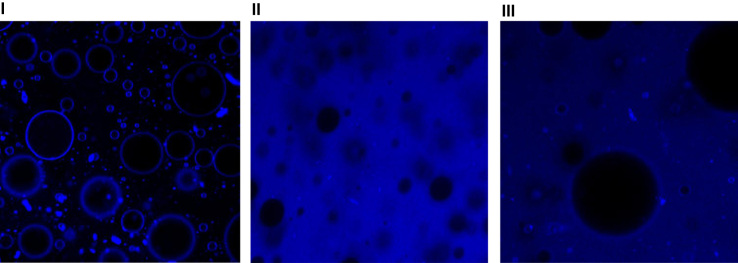
Confocal scanning light microscopy of emulsions in which the lipid phase was stained with Nile blue: I) System C (0.250% xanthan gum and 44.0% oil); II) System B (1.000% xanthan gum and 11.0% oil) and III) System E (1.000% guar gum and 44.0% oil).

The data reported include information about the physical stability of oil in water emulsions with different compositions (Table 1) through the estimation of Span and Specific Surface Area (Table 2) and the recorder of confocal scanning laser microscopy images (Fig. 1).

## 2 Experimental design, materials and methods

Oil in water emulsions were prepared using a high speed homogenization and their composition is given in Table 1. Emulsion preparation was described in the research article [1].

### 2.1 Span and Specific Surface Area

Span and SSA of emulsions was determined by light scattering using a Mastersizer 2000 with a Hydro 2000 MU as dispersion unit (Malvern Instruments, Worcestershire, United Kingdom). A refractive index of 1.473 for the corn oil phase and its absorption parameter (0.001) was used. Determinations were made after 24 h of emulsification and after 7 days of storage. Span is a measure of polydispersity of oil droplets and is defined as:

$$P=\frac{\left(d_{09}-d_{01}\right)}{d_{05}}$$

being *d*_01_, *d*_05_ and *d*_09_ the fractions of droplets with diameters smaller than 0.1, 0.5 and 0.9, respectively [2], [3]. The SSA expresses the ratio between the total area of the droplets and their total weight. It can be estimated as:

$$\mathrm{SSA}=\frac{6\emptyset }{D_{32}}$$

where *ϕ* is the volumetric fraction and *D*_32_ is Sauter diameter. Data reported were the mean of ten determinations made on two different emulsions of identical composition. Some emulsions were inoculated with *Zygosaccharomyces bailii.* Results obtained are shown in Table 2.

### 2.2 Confocal scanning laser microscopy

The microstructure of the emulsions was evaluated by placing aliquots of 10 μL emulsion (without prior dilution) on a slide. The coverslips (22×22 mm) -without sliding- were carefully placed to not induce coalescence of the oil droplets. Then, emulsions were observed with a laser confocal microscope (Model FV 300, Olympus, UK), equipped with a He–Ne laser (543 nm). A PLAN APO 60X objective and 2.5X digital zoom was used. Digital images in TIFF format were purchased in 1024×1024 pixel resolution. The lipid phase was labeled with Nile blue (aqueous solution 0.1% p/v, *λ*_exc_=635 nm) [4], [5]. Some of the images obtained are shown in Fig. 1.

## Figures and Tables

**Table 1:** Concentrations of corn oil, xanthan and guar gum in model systems.

**System**	**Xanthan gum** (wt%)	**Guar gum** (wt%)	**Corn oil** (wt%)
A	0.250	0.000	11.0
B	1.000	0.000	11.0
C	0.250	0.000	44.0
D	1.000	0.000	44.0
E	0.000	1.000	44.0

**Table 2:** Span and Specific Surface Area (m^2^/g) of inoculated and non-inoculated emulsions after one day and seven days of storage at 25 °C.

**System**	**Span±standard deviations**			
	**Non-inoculated**		**Inoculated**	
	Day 1	Day 7	Day 1	Day 7
A	1.886±0.009	1.941±0.064	1.902±0.001	1.908±0.036
B	1.440±0.016	1.430±0.002	1.433±0.001	1.435±0.003
C	1.552±0.016	1.527±0.008	1.442±0.009	1.465±0.026
D	1.690±0.026	1.676±0.007	1.685±0.036	1.686±0.001
E	0.689±0.012	0.790±0.006	0.762±0.079	0.690±0.001
**System**	**Specific Surface Area±standard deviations (m**^**2**^**/g)**			
	**Non-inoculated**		**Inoculated**	
	Day 1	Day 7	Day 1	Day 7

A	1.015±0.007	1.01±0.001	0.999±0.002	0.965±0.012
B	1.305±0.106	1.215±0.007	1.225±0.021	1.275±0.035
C	0.623±0.006	0.607±0.002	0.575±0.004	0.574±0.006
D	0.592±0.006	0.635±0.009	0.614±0.059	0.560±0.001
E	0.064±0.001	0.064±0.001	0.067±0.001	0.064±0.001
